# Supplementary material for: Are carbon emissions trading and green financial instruments synergistic? -Comprehensive quantitative research based on content analysis
Source: PLoS One. 2024 Mar 7;19(3):e0298601. doi: 10.1371/journal.pone.0298601 (PMC10919601; doi:10.1371/journal.pone.0298601)
Supplement: S1 Data — (ZIP) [file pone.0298601.s001.zip › Data/地级市Z_F工作报告.zip!/║╙▒▒/║¬╡a╩╨/└┤╫╘╣1⁄2╓┌║┼í╛┬φ┐╦╩2╛▌═°í┐_2014.docx]

各位代表:

现在，我代表邯郸市人民政府向大会作工作报告，请予审议，并请市政协委员和列席会议的同志提出意见。

一、2013年工作回顾

2013年是本届政府的开局之年，也是我市发展形势极为复杂严峻的一年。一年来，市政府在省委、省政府和中共邯郸市委的坚强领导下，在市人大、市政协的监督支持下，紧紧围绕建设宜居宜业宜游富强邯郸、美丽邯郸的战略目标，致力稳增长、调结构、优环境、惠民生，较好完成了市十四届人大一次会议确定的工作任务。

--积极应对经济下行压力，稳中有进、进中提质的发展态势进一步明显。全市生产总值完成3061.5亿元，增长7.3%;全社会固定资产投资完成2758.9亿元，增长15.7%;社会消费品零售总额完成1094.2亿元，增长13.6%。在保持经济平稳发展的同时，更加注重做好质量效益、基础后劲、惠及民生的工作。公共财政预算收入完成172.7亿元，占全部财政收入比重达到57.3%，提高1.2个百分点;全部财政收入和公共财政预算收入中税收占比分别达到81.2%和67.2%，提高2个和4.2个百分点。采取"三集中"的办法推进重点项目建设，武安新能源一期、戴克电器、恒博新材料等一批产业项目相继投产，邯黄铁路、机场二期等重大基础设施建成投运，南水北调邯郸段具备通水条件。新增规模以上工业企业240家，规模以上工业增加值完成1273.6亿元，增长8%。认定科技型中小企业1202家，居全省第一。城镇居民人均可支配收入23936元，农民人均纯收入9542元，分别增长10.1%和13%，高于生产总值增速2.8个和5.7个百分点。

--深入调整产业结构，经济转型升级步伐进一步加快。立足"有中生新"抓升级，改造提升传统产业，钢铁产业整合重组已经破题，实施方案得到省政府肯定，永年县、峰峰矿区整合工作进展顺利，武安明芳公司牵头达成5家企业的并购协议。完成技改投资883.9亿元，增长19.9%。坚持"无中生有"促转型，培育壮大战略性新兴产业，规模以上装备制造业增加值完成128.5亿元，增长23.5%，成为工业第二大行业。高新技术产业增加值增长10.6%，我市列入全国首批新能源汽车推广应用城市，奥玻集团荣获国际"碳金生态实践奖"。"三年千项"工程累计新上项目876个，完成投资1723亿元。加快发展现代服务业，邯郸国际陆港跻身省级物流产业聚集区，广府古城、七步沟获批4A级景区，旅游业总收入增长29.6%。服务业增加值完成1079.7亿元，增长8.2%。加大科技创新力度，荣获国家科技进步二等奖2项，新增国家高新技术企业24家。邯郸经济开发区晋升为国家级经济技术开发区，冀南新区获批国家级新型工业化产业示范基地，节能环保产业园列入省5家专业园区之一。

--扎实做好"三农"工作，农业基础地位进一步巩固。粮食总产555.6万吨，荣获"全国粮食生产先进市"。出台推进农业产业化经营、加快农村土地流转4个专件，在全省率先推出"金财通"贷款模式。新增土地流转面积27.9万亩，流转率达到18.8%，提高3.1个百分点，规模经营面积111.7万亩。农业产业化经营率预计达63.8%，提高1.5个百分点。邯郸农业科技园区获批国家级园区。新增节水灌溉46万亩。太行山前土地整治示范区项目竣工，整理规模107万亩。农村面貌改造提升行动成效明显，217个省级重点村实施3844个实事项目。20万人实现稳定脱贫。

--注重统筹城乡协调发展，城镇面貌进一步改善。着力构建中心城市、次中心城市、特色县城、建制镇、新型农村社区良性互动的城镇体系，城乡一体化进程不断加快。规划体系日臻完善，编制完成中心城市空间发展战略规划、历史文化名城保护规划和县城规划。市级城建工程开工313项，完成投资434亿元。东区建设定位进一步明确，招商引资和西军师堡村征迁、"两违"整治稳步推进，市客运枢纽中心开工建设，丛台路、联纺路东延竣工通车。滏阳河改造提升工程一期通航，串城街征迁工作基本完成。新建、改建城市路桥工程39项。供热、供水、供气和污水处理等配套设施进一步完善。城市轨道交通项目谋划启动，邯郸火车站改扩建工程开工。主城区回迁房开工10470套(户)、竣工15351套(户)。县城建设全面加快，开工重点工程441项，完成投资620亿元。城市管理水平不断提高，环卫管理体制日益完善。

--加快推进改革开放，发展活力和动力进一步增强。重点领域改革步伐加快，市属国企改革攻坚扫尾全面完成，县级公立医院综合改革试点扩大到12个县(市、区)，积极落实"营改增"，市级食品药品监管机构改革基本完成。全方位扩大开放，通过廊坊、上海、厦门和邯郸4次集中招商活动，签约合同项目221个，总投资4238亿元，分别增长45%和120%。引进战略投资者成效明显，与央企新签订协议21个，总投资621亿元，新增中铁建、中钢研等合作央企10家，与新兴际华、中冶科工、北京铁路局等驻邯央企的战略合作进一步深化，土耳其佐鲁、五粮液、卓达等一批国内外知名企业入驻。实际利用外资8.8亿美元，增长10.4%。金融工作日趋活跃，3家银行入驻邯郸，魏县爱美森在香港创业板上市，新增7家挂牌企业，邯郸银行实现县域全覆盖，发行城投、交建、中小企业集合债券39.6亿元，首支股权投资基金投放。民营经济加快发展，新增市场主体3万多户，完成增加值1980.3亿元，增长8.5%。

--大力改善"两个环境"，区域竞争优势进一步彰显。在发展环境上，突出压审批、降收费、减时限、少检查、提服务、查案例，市本级行政许可事项由119项削减为95项，非行政许可审批事项由97项下降到63项，取消和免征行政事业性收费40项，公开处理典型案件33起。在生态环境上，围绕打好大气污染防治攻坚战，落实削煤、压钢、减排、控车、降尘等措施，淘汰改造燃煤锅炉161台，削减煤炭消费80万吨;拆除高炉3座，压减生铁产能131万吨;实施减排工程609项，关停取缔各类重污染小企业2620家;淘汰黄标车9万辆。启动重污染天气Ⅲ级应急响应4次，依法查处环境违法案件10起。彻底清理东武仕水库网箱养鱼，我市列入全国水生态文明城市建设试点。启动创建国家森林城市，打造"绿美邯郸"，完成造林46万亩。去年12月份，细颗粒(PM2.5)平均浓度比1月份下降17.4%。预计全市单位生产总值能耗下降2.8%，化学需氧量、二氧化硫、氨氮和氮氧化物均可完成省定减排目标。

--切实保障和改善民生，人民群众的幸福指数进一步提高。财政用于民生支出219亿元，增长8.6%，95件实事如期完成。城镇新增就业10.8万人，新增农村劳动力转移就业8.9万人。城乡居民社会养老保险参保率达到98.6%，企业退休养老金待遇人均月增188元，城乡低保月人均补差分别提高25.9元和17.3元，农村"五保"集中供养率达到60%，80岁以上高龄老人生活补贴制度实现全覆盖。新建农村幸福院、社区居家养老服务中心569个。保障性安居工程开工16849套(户)、竣工28070套(户)，位居全省前列。解决了633个村、77万人的饮水安全问题。新建标准化中小学50所，省级示范性幼儿园达到35所，改造中小学校舍645.9万平方米。新建改建2所县级医院、31所乡镇卫生院和3871个村卫生室，城市社区卫生服务中心覆盖率达到100%。新农合参合率达到99.1%，居全省第一。文化建设实现突破，荣获"群星奖"、"文华奖"、"梅花奖"、"法国国家沙龙展金奖"等一批重大奖项，"十全十美"图乘"神十"飞天，赵王城遗址获国家考古遗址公园立项，新增全国重点文物保护单位16处。全民健身运动蓬勃开展，滏阳河、沁河沿岸百里健身长廊被评为国家精品体育公园。社会管理不断创新，成功应对水污染等突发事件，注重从源头上解决信访问题，在保障建设领域农民工工资、处置医患纠纷等方面建章立制，安全生产形势稳定好转，食品药品第三方监测深入实施，构建"平安邯郸"网格化巡防体系，人民群众安全感明显增强。

--全面加强政府自身建设，政府公信力和执行力进一步提升。围绕打造诚信政府，正确处理继承、发展、创新的关系，保持政策稳定性和连续性，在事关群众切身利益的建筑、食品药品安全等领域实行"黑名单"制度，以政府诚信带动社会诚信和商务诚信。围绕打造法治政府，注重学法用法，坚持依法行政，完成重点立法项目6件，公开政务信息4.4万条。自觉接受人大法律监督和工作监督、政协民主监督和社会监督，实行邀请市人大、市政协负责同志列席市政府常务会议制度，办理人大代表建议316件、政协提案557件，按时办复率均为100%。围绕打造廉洁政府，严格落实中央"八项规定"和省市有关要求，深入推进正风肃纪行动，全面开展违规公务用车、超标办公用房、"吃空饷"人员清理。加大审计监督、行政监察力度，政府廉政建设不断加强。

同时，国防动员和双拥共建扎实开展，民族宗教、外事侨务、广播影视、新闻出版、邮政通信、妇女儿童、防震减灾、人防、统计、物价、气象、档案、老龄、援疆、残疾人等工作，都取得了新成绩。

各位代表!以上成绩来之不易。这是省委、省政府和中共邯郸市委正确领导的结果，是市人大、市政协监督支持的结果，是全市人民扎实苦干的结果。在此，我代表市政府，向全市人民，向各位人大代表、政协委员，向各民主党派、工商联、无党派人士和人民团体，向驻邯人民解放军、武警官兵和政法干警，向中直、省直驻邯各单位，向各位老领导，向所有关心支持邯郸发展的同志们、朋友们，表示崇高的敬意和衷心的感谢!

在肯定成绩的同时，我们也清醒地看到，面对复杂多变的宏观经济形势，特别是受我市产业结构偏重、节能减排约束、化解过剩产能、支柱行业市场低迷等因素影响，去年全市生产总值、规模以上工业增加值、社会消费品零售总额等指标没有达到预期目标，尤其是全部财政收入下降8.5%，进出口总值下降3.2%。经济社会发展中还存在着一些突出问题:部分企业生产经营困难，有的还没有摆脱亏困局面;服务业比重偏低，科技创新能力不强;节能减排和生态环境治理任务艰巨，提高大气质量迫在眉睫;一些部门服务意识不强，办事效率不高，发展环境仍需进一步优化;在改善民生方面还有许多亟待解决的问题。对此，我们一定本着对人民高度负责的精神，采取有力措施，切实予以解决。

立足新的起点，我们面临着诸多发展机遇和有利条件。党的十八届三中全会和省委八届六次全会，描绘了全面深化改革的"路线图"，有利于激发市场活力和内生动力。我市相继纳入中原经济区规划、首都经济圈规划、全国老工业基地调整改造规划和资源型城市可持续发展规划，四大国家级战略的叠加效应，有利于我市借势中原、对接京津、融入沿海，催生新一轮又好又快发展。一批铁路、陆港、高速公路、水利、电力等重大工程建成投用，有利于构筑可持续发展的基础支撑。市委[1] 八届五次全会开启了建设宜居宜业宜游富强邯郸、美丽邯郸的新征程，全市干部群众干事创业的激情更加高涨，有利于凝心聚力，更好地把各项事业推向前进。机遇千载难逢，发展时不我待。我们一定准确把握形势，乘势而上，主动作为，奋力开创经济社会发展的全新局面。

二、2014年目标任务和工作重点

今年政府工作的总体要求是:全面贯彻落实党的十八届三中全会、省委八届六次全会和市委八届五次全会精神，坚持稳中求进的工作总基调，把改革创新贯穿于经济社会发展各个领域各个环节，把新型城镇化作为发展的重大潜力，全力稳增长、调结构、促改革、提效益、惠民生，突出抓好项目建设、环境建设、城乡建设、平安建设，推动经济持续健康发展、生态明显改观、社会和谐稳定，加快建设宜居宜业宜游的富强邯郸、美丽邯郸，不断夺取全面建成小康社会新胜利。

全市经济社会发展的主要预期目标是:全市生产总值增长7.5%，公共财政预算收入可比增长9%，全社会固定资产投资增长17%，社会消费品零售总额增长13%，进出口总值增长6%，实际利用外资增长8%。单位生产总值能耗下降2.8%，化学需氧量、二氧化硫、氨氮、氮氧化物排放量和细颗粒物浓度完成省定目标。居民可支配收入增长9%，居民消费价格涨幅控制在3.5%左右。城镇登记失业率控制在4%以内，人口自然增长率控制在8.6‰以内。

按照上述要求，重点把握好四个方面:一是更加注重战略全局思维。围绕建设宜居宜业宜游富强邯郸、美丽邯郸的战略目标，按照"中原经济区内具有重要影响力的中心城市、中原经济区与环渤海等经济区域合作交流的北部门户"的发展定位，谋划推进经济社会发展各项工作，形成全市"一盘棋"的工作格局。二是更加注重推进科学发展。转变经济发展指导思想，冲破传统思维模式和路径依赖，坚定不移地转方式、调结构，把经济工作的着力点放在提升质量效益上来，谋求更高质量、更好效益的绿色崛起。三是更加注重改革开放引领。充分释放改革、开放、创新的最大红利，靠改革增动力，靠开放添活力，靠创新谋出路，推动经济更有效率、更加公平、更可持续发展。四是更加注重打造"两个环境"。不断优化发展环境，致力改善生态环境，让市场活力竞相迸发、先进要素竞相涌入，让邯郸的天更蓝、水更清、地更绿、环境更宜居。

重点做好九个方面的工作:

(一)着力加强重点项目建设，在提高发展质量和效益上求突破。坚持把项目建设作为核心抓手，保持投资强度，优化投资结构，提高投资质量，靠优质增量促进转型升级。

实施"1155"工程。全市抓好100个在建项目，推进100个前期项目，核准备案亿元以上项目500个，市级直抓投资50亿元以上的大项目。加大谋划储备力度，引上一批高精尖项目，培育国内领先、世界一流的领军企业。强力推进重大基础设施项目建设，确保邯长邯济铁路扩能改造、邯大高速等工程竣工投用，积极推进风力发电、光伏地面电站、天然气利用等项目建设。

打造项目聚集平台。加快冀南新区开发建设，重点推进节能环保产业园、台湾产业园等"园中园"建设，打造全国重要的先进制造业基地。支持邯郸经济技术开发区用好"国字号"招牌，加快扩区步伐，做大经济总量，早日建成科技领先、实力雄厚的多功能综合性园区。抓住省整合产业园区的机遇，抓好现有园区规范提升，支持临漳县、峰峰矿区、邯山区争创省级开发区，搭建一流平台，聚集优质产业。

完善项目管理机制。把好项目准入关，严格项目环评、能评、安评等，坚决卡死影响环境的项目。加强项目用地管理，70%以上建设用地指标用于工业项目。建立项目预警和退出机制，实行土地指标动态管理，严肃查处违法用地行为，对圈而不建的，坚决依法收回土地。完善项目推进机制，开工项目看进度，在建项目看竣工，竣工项目看效益，把项目建设的成效体现在就业增加和税收增长上。

(二)着力加快土地流转，在发展现代农业上求突破。坚持把农村土地流转作为保障粮食安全、发展现代农业、激发农村活力的切入点，加快农业现代化进程，让农业强起来，让农村美起来，让农民富起来。

搞好确权颁证。按照依法、自愿、有偿的原则，落实集体所有权、稳定农户承包权、放活土地经营权，全面推进农村土地承包经营权确权颁证工作。完善土地流转服务平台，探索建立农村产权交易中心，鼓励承包经营权在公开市场流转。

培育经营主体。按照"土地流转提高效益、农民流动提高素质"的思路，鼓励发展、大力扶持家庭农场、专业大户、农民合作社、产业化龙头企业等新型主体，发展规模经营，形成一批千亩以上土地流转示范方。积极推进集体林地流转。全市土地流转面积达到193.6万亩，流转率达到21.8%。以现代农业园区建设带动土地流转，打造一批高标准的示范区。

强化政策扶持。市县财政安排专项资金，对流转面积千亩以上、从事粮棉生产的规模经营主体给予奖补。整合财政涉农资金，集中扶持一批市场前景好、带动能力强的龙头企业。全市农业产业化经营率提高1.5个百分点。

高度重视粮食生产，建设150个万亩高产示范方，实施"棉麦双丰"工程，支持蔬菜、奶业等产业发展。加强农田水利建设，引供地表水5亿立方米，发展节水灌溉66万亩。

(三)着力抓好钢铁产业整合重组，在工业转型升级上求突破。坚持"有中生新、无中生有"两手抓，改造提升传统产业，培育壮大新兴产业，加速新型工业化进程。

加快钢铁产业整合重组。坚持化解产能、节能减排、大气治理"三合一"集中推进，加快武安市、永年县、峰峰矿区三大重点区域整合重组步伐，做到结构调整、职工安置、债务化解"三到位"。综合运用市场、法律、行政、标准等手段，特别是用好环保法、水法和土地法，倒逼过剩产能退出市场。全市压减生铁产能284万吨、粗钢产能211万吨。坚持走质量、品种、效益之路，以市场为导向，调整优化钢铁产业、产品结构。搞好产需对接，延伸产业链条，发展吃钢产业，全市钢铁就地消化率提高到40%左右。

培育战略性新兴产业。重点发展新材料、先进装备制造产业，培育发展节能环保、电子信息、航空航天、新能源、生物等产业，加快718所科研成果、硅谷化工特种纤维产业化步伐，推进新能源汽车发展和动力电池产业化建设。力争战略性新兴产业增加值增长14.5%。

扶持壮大规模企业。集中扶持10家行业龙头企业、10家高成长性企业，带动产业转型升级。深入实施"三年千项"工程，确保规模以上企业累计新上项目1000个，完成投资1800亿元。新增规模以上工业企业185家以上。全市规模以上工业增加值增长9.5%。

提高科技创新能力。加强企业自主创新能力建设。推动各类创新要素向企业聚集，引导企业加大研发设计投入，加强与高等院校、科研单位的合作，新建企业研发机构280家以上。推进国家级创新型产业集群建设，发展高新技术企业15家，培育科技型中小企业500家。

(四)着力提升发展规模和水平，在壮大现代服务业上求突破。坚持把发展现代服务业作为建设中心城市、北部门户的重要支撑，聚集更多的人流、物流、信息流、资金流。全市服务业增加值增长8.5%。

大力发展商贸物流业。提升商贸服务业，完善中心城区商贸布局，引进家乐福、奥特莱斯等知名企业和新型业态。编制出台《现代服务业发展三年规划》，加快发展现代物流业，以推进与马来西亚隆基马中公司的战略合作为契机，着眼发展大物流，谋划建设现代物流园区。建设武安保税物流中心、冀中能源国际港保税区，确保国际陆港开港运营，提升邯郸辐射带动力。同时，发展服务外包、电子商务等新兴业态，加快发展健康服务业，培育服务业新亮点。

做大做强文化旅游业。牢固树立大旅游理念，整合旅游资源，走市场化运作的路子。打造以主城区为中心的"一小时黄金旅游圈"，抓好凤凰山-溢泉湖、邺城、紫山、九龙山等36个旅游重点项目建设，支持娲皇宫、广府古城争创5A级景区。完善吃住行游购娱等要素，构建高标准旅游产业体系，提升邯郸旅游影响力。

繁荣活跃现代金融业。创新金融产品，促进信贷扩规模、优结构、提效益。加大企业上市力度，每个县(市、区)至少培育3家上市公司，全市新增上市挂牌企业10家以上。积极引进金融机构，支持邯郸银行增设域外分支机构。引导支持各类投资公司、专业合作社健康发展，加强规范管理，防范和化解金融风险。

(五)着力强化大气整治，在生态文明建设上求突破。坚持把大气污染整治作为重中之重，以壮士断腕的勇气，以科学严谨的态度，背水一战，下大力治理生态环境。

强化政策激励，运用科学方法，综合整治大气环境。强力淘汰落后产能，搞好源头治理，削减煤炭消费427万吨。开展钢铁、焦化、水泥、电力、玻璃五个行业大气污染治理攻坚行动，实施359项减排项目，确保减排除尘设施达标运行。淘汰黄标车3.1万辆以上。治理建筑施工扬尘，推行新建住宅全装修、工地路面全硬化等"十个100%"。坚持大气污染"零容忍"，从严查处各种环境违法行为，确保大气环境每年都有好转。

大力推进"绿美邯郸"攻坚行动，完成造林绿化52万亩，森林覆盖率达到26%以上。以对子孙后代和长远发展高度负责的态度，全力打造水生态，严控南水北调沿线各类污染物排放，确保滏阳河各跨界断面水质达到水功能区划标准。加大地下水污染防治力度，加强饮用水源地保护，做好水源涵养工作。强化土地污染防治和矿山治理，搞好生态环境保护和修复。

各位代表，我们头顶同一片蓝天，脚踏同一方热土，保护和修复生态环境是每一个邯郸人的共同责任。让我们心连心，手牵手，从我做起，从身边做起，从点滴做起，共建天蓝水净、地绿山青的美好家园!

(六)着力推进新型城镇化，在加快城乡一体化上求突破。坚持把新型城镇化作为强大引擎，突出以人为本，完善功能布局，传承历史文脉，走出一条独具特色的城镇化道路。完善规划布局。做大做强中心城市，推进中心城区与周边县城"1+8"组团式发展，避免"摊大饼"。按照"东区引领、南北拓展、老城提升"的思路，加快中心城区建设。完善县城和重点镇规划。维护规划严肃性，做到一张蓝图干到底。

提升城市承载能力。突出抓好东区开发建设，加快路网建设和村庄征迁，完成市客运枢纽中心主体工程，实施教育园区、体育中心、传媒中心、科技中心、青少年活动中心等项目。搞好串城街历史文化街区修缮整治，推进滏阳河整体改造提升，加快黄粱梦滞洪区、支漳河等生态水系建设，彰显城市文化底蕴和生态魅力。实施旧改回迁、城市道路、配套设施等一批便民项目，对92条小街巷全部整治。优先发展城市公共交通，加快推进城市轨道交通项目，谋划邯峰快速路建设，推动基础设施向周边延伸，催生同城效应。

强化精细化管理。有序推进农业转移人口市民化，加快户籍制度改革，稳步推进城镇基本公共服务常住人口全覆盖，让农民进得来、留得住、过得好。完善城市管理体制,加强市容市貌治理，解决占道经营、破墙开店、马路市场、噪音污染等问题，规范物业管理市场。完成数字邯郸地理空间框架建设，提高数字城管水平，加快建设智慧城市。

推进县城扩容升级。树立建设中小城市的理念，引导产业和人口向县城聚集。科学布局县城和园区，完善基础设施，建设一批城市综合体、商贸物流等项目，更好地承接农村转移人口。适时调整行政区划，不断拓展发展空间。同时，加快发展特色鲜明的建制镇，在县城周边鼓励建设新型农村社区，引导农民转变生产生活方式，加快城乡一体化进程。以"房水路绿厕能"为重点，扎实推进农村面貌改造提升，建设升级版的现代新农村。

(七)着力深化改革扩大开放，在增创发展新优势上求突破。坚持把改革开放作为解难题、活全局的根本途径，加快形成有利于科学发展的体制机制和大开放格局。

全面深化各项改革。深化行政管理体制改革，落实好国家、省取消下放的行政审批事项。大力推进商事登记制度改革，实行投资主体"零限制"、注册登记"零收费"。积极稳妥地推进政府向社会力量购买公共服务，逐步实现由"花钱养人"向"花钱办事"的转变。围绕赋予农民更多财产权利，全面深化农村改革。推进财税金融体制改革，防范和化解政府债务风险。积极探索股权交易融资，支持具备条件的民间资本投资金融机构。统筹推进科技、文化、教育等社会事业改革，充分激发发展活力。

提高对外开放水平。树立世界眼光和全球思维，瞄准产业高端和发展前沿，积极引进战略投资者，以大开放带动大发展。紧盯京津、长三角、珠三角等重点区域，主动承接资金、产业、人才、成果转移。加大央企对接力度，实施大企业、大集团战略。注重外贸出口多元发展，鼓励邯钢、峰峰集团、晨光集团等企业以多种方式"走出去"，建设境外基地。扩大钢铁、纺织服装、机电等产品出口，全市进出口企业达到1000家以上。

加快发展民营经济。落实鼓励民营经济发展的各项政策，制定《推进全民创业激活市场主体的实施意见》，坚持权利平等、机会平等、规则平等，进一步消除市场准入的各种隐性壁垒，完善中小企业社会化服务体系，促进民营经济健康发展。民营经济增加值增长8.5%。

大力优化发展环境。集中整治企业周边环境，从严打击欺行霸市、寻衅滋事等违法犯罪行为。推进职能部门和窗口单位提质提效，充分发挥企业110作用，严肃查处"中梗阻"、设障刁难、吃拿卡要等问题。谁破坏邯郸的发展环境，就砸谁的"饭碗子"!

(八)着力统筹区域发展，在壮大县域经济上求突破。坚持把县域经济作为邯郸绿色崛起的基石，按照区域经济

一体化的理念，优化产业布局，加大基础设施投入，理顺财税划分体制，促进县域经济更好发展。

围绕西部提升，支持武安市、峰峰矿区、永年县加快钢铁整合重组，开展二次创业，带来产业转型升级脱胎换骨的变化。支持磁县、涉县走多元发展之路，大力发展先进装备制造、精细煤化工、文化旅游等新兴产业，培育新的经济增长点。

围绕中部繁荣，支持丛台区、邯山区、复兴区和邯郸县，发展总部经济、楼宇经济，建设城市综合体、大型商贸服务中心和现代物流园区，打造聚集高端产业、财富竞相涌流的高地。

围绕东部振兴，支持曲周县自行车配件、魏县再生物资、邱县棉纺、鸡泽县辣椒和铸件、馆陶县禽蛋和轴承、临漳县木炭等家庭作坊式企业，依靠科技进步、规范管理，实现上档升级，培育富民兴县的特色产业集群。支持成安县、广平县发挥工业基础优势，对标行业高端，提升管业、汽车等支柱产业的核心竞争力。支持大名县延伸特色产业链条，打造食品工业强县。支持肥乡县加快发展城郊经济，实现与主城区错位发展。加大扶贫攻坚力度，确保15万人稳定脱贫。

(九)着力创新社会治理，在保障和改善民生上求突破。坚持把民之所望作为施政所向，把政府职能更多地转向公共服务和社会管理，努力让人民生活得更加殷实、更加幸福、更有尊严。

推进基本公共服务均等化。围绕少有所学、壮有所为、老有所养、老有所乐，提供更多优质公共服务和产品。坚持就业为本，重点抓好高校毕业生就业和化解过剩产能中出现的失业人员再就业，统筹抓好农村转移劳动力、城镇困难人员、退役军人就业，推动实现更高质量的就业，新增城镇就业10万人以上，农村劳动力转移就业4万人以上。以办好人民满意的教育为目标，学前教育抓普惠、义务教育促均衡、高中教育提质量、职业教育重实用、高等教育创特色，实施第二期学前教育行动计划，建成50所农村标准化中小学，强化中小学安全管理及周边环境治理，加快主城区西部省级示范性高中建设，建设职业教育实训基地。加快医疗卫生事业发展，全面深化县级公立医院改革，推进基层医疗机构综合改革，加强重大疾病和传染病防控，建设提升1000个健康小屋。完善养老服务体系，新建农村幸福院214所、社区居家养老服务中心114个。大力发展文化事业和文化产业，搞好非物质文化遗产保护与传承，办好第二届中国吹歌节、第九届中原民间艺术节。完善全民健身体系，举办第十二届国际太极拳运动大会。稳定低生育水平，提高出生人口素质。统筹推进军民融合式发展、新闻出版广电、老龄妇女儿童、气象防震减灾、民族宗教、邮政通信、人民防空等各项事业，为城乡居民提供多样化的公共服务。

多办利民惠民实事。财政用于民生投入200亿元以上，实施好十大工程96件实事。坚持保基本、兜底线、促公平、可持续，健全覆盖城乡居民的社会保障体系，提高新农合、城镇居民医保补助标准和报销水平，实现人人享有基本社会保障。制定完善失地农民保障措施，统筹解决好就业、养老等问题。健全社会救助体系，积极发展社会福利和慈善事业。加快住房供应体系和保障性安居工程建设，新开工保障性住房15500套(户)，竣工28000套(户)，分配入住27100套(户)。实施农村饮水安全工程，解决521个村、64万人饮水安全问题。

加强和创新社会治理。适应社会发展新形势，深入研究社会治理新问题，构建多元化社会治理结构，注重从源头上建章立制，实施长效管理，维护社会公平正义。完善社会稳定风险评估机制，扎实做好信访工作，预防和妥善处置群体性事件。健全公共安全应急机制，提高突发事件应急处置能力。严格落实安全生产责任制，坚决遏制重特大事故发生。健全食品药品监管体系，确保全市人民饮食用药安全。依法严厉打击违法犯罪行为，构筑"平安邯郸"立体化社会治安防控体系，营造和谐稳定的社会环境。

各位代表!面对加快转型升级、实现绿色崛起的繁重任务，我们一定以奋发有为的精神状态，聚精会神谋发展，紧盯目标抓落实，不断谱写邯郸各项事业的崭新篇章!

三、加强政府自身建设

打造诚信政府、法治政府、廉洁政府，是本届政府的庄严承诺。我们一定以开展党的群众路线教育实践活动为契机，反"四风"、转作风，做到为民、务实、清廉，努力让人民群众满意。

(一)转变职能，恪尽职守。把市场在资源配置中起决定性作用和更好发挥政府作用有机结合，把政府角色定位在为民上，把政府职能转变到服务上，寓管理于服务之中,在服务中履职尽责。开展政府机关标准化建设，以企业和群众需求为导向，创新政府服务模式，实现从"审批型"向"服务型"转变，努力营造高效、便民、透明的政务环境。

(二)民主决策，依法行政。围绕推进依法决策、科学决策、民主决策，建立健全公众参与、专家论证和集体决定相结合的行政决策机制，完善决策跟踪反馈、责任追究等制度，使各项决策更加反映民意、集中民智、符合民愿。把遵守宪法和法律作为施政的根本原则，推进行政权力公开规范运行，把政府工作纳入法治轨道。自觉接受人大法律监督、政协民主监督，充分听取各民主党派、工商联、无党派人士和各人民团体的意见，认真办理人大代表建议和政协提案。主动接受舆论监督和社会公众监督，保证人民群众的知情权、参与权、表达权和监督权。

(三)深入一线，攻坚克难。坚持把经济社会发展一线作为政府工作的主战场，从市政府班子带头做起，加强调查研究，坚持问题导向，深入项目工地、企业车间、田间地头，解难题、办实事。面对工业转型、大气整治、改革攻坚、社会治理等繁重任务，迎难而上，勇于担当，以踏石留印、抓铁有痕的作风，啃硬骨头，打攻坚战，致力破解瓶颈制约，带动活跃发展全局。

(四)勤政为民，清正廉洁。坚决执行中央关于改进工作作风、密切联系群众的八项规定，带头落实中央、省、市关于公务接待、办公用房、公务用车、差旅费等各项要求。厉行勤俭节约，严控"三公"经费支出，降低行政成本，把更多财力用在促发展、惠民生上。严格落实党风廉政建设责任制，深入推进廉政风险全方位预警防控系统，加强政府支出项目和政府规章制度廉洁性评审，从源头上预防腐败，做到干部清正、政府清廉、政治清明。

各位代表!建设宜居宜业宜游的富强邯郸、美丽邯郸，在古赵大地春潮涌动。美好的蓝图鼓舞着我们，神圣的使命感召着我们，人民的期待激励着我们。让我们在省委、省政府和中共邯郸市委的坚强领导下，惟公惟民不动摇，尽职尽责不惜力，克勤克俭不懈怠，上下一心，拼搏实干，为加快推进邯郸复兴伟业、全面建成小康社会而努力奋斗!
